# Rat salivary gland ligation causes reversible secretory hypofunction

**DOI:** 10.1111/j.1365-201X.2006.01662.x

**Published:** 2007-03-01

**Authors:** G H Carpenter, S M Osailan, P Correia, K P Paterson, G B Proctor

**Affiliations:** Salivary Research Unit, Floor 17, Guy's Tower, King's College London London, UK

**Keywords:** acinar, calcium, ductal, hypofunction, inflammation, ligation, saliva, salivary

## Abstract

**Aim:**

To determine the influence of inflammation on salivary secretion. Secretion by salivary glands involves interactions between nerves, blood vessels and salivary cells. The present study investigated the effects of inflammation on rat submandibular gland function following acute ductal obstruction.

**Methods:**

Under recovery anaesthesia a metal clip was placed on the main duct of the submandibular gland. After 24 h salivary secretion was evoked by nerve and methacholine stimulation. For recovery experiments the clip was removed after 24 h and the animal left to recover for 3 days when salivary function was again assessed.

**Results:**

By 24 h of obstruction an inflammatory infiltrate had developed within the obstructed gland and stimulated salivary flows were just 20% of the normal secretion, whilst protein secretion and ion reabsorption were also severely impaired. If ductal obstruction was removed after 24 h the salivary function returned to normal after 3 days of recovery. *In vitro* analysis of cells from 24-h ligated glands revealed normal changes in intracellular calcium (the main secondary messenger involved in fluid secretion) in response to methacholine stimulation. Protein secretion from isolated cells indicated some changes in particular to methacholine-induced protein secretion although a significant protein secretion was still seen in response to isoprenaline – the main stimulus for protein secretion.

**Conclusion:**

This report demonstrates reversible salivary inhibition associated with an inflammatory infiltrate within the salivary gland.

Saliva forms a film over teeth and mucosal surfaces in the mouth (Sas & Dawes 1997) and performs a number of functions, including lubrication, prevention of soft tissue desiccation and tooth demineralization and the maintenance of an ecological balance (Amerongen & Veerman 2002). Saliva is secreted by three pairs of major salivary glands – parotid, submandibular and sublingual – as well as numerous other minor salivary glands located around the mouth. Salivary gland secretion of fluid and proteins is controlled by autonomic nerves and once these nerves have been sectioned secretion ceases almost entirely. Parasympathetic nerve-mediated stimuli and the release of acetylcholine play the principal role in activating salivary acinar cell fluid secretion. Concomitantly glandular blood flow is increased via cholinergic, peptidergic and nitric oxide-mediated vasodilatation (Anderson & Garrett 1998, Edwards 1998). In rat parotid and submandibular glands release of noradrenaline from the sympathetic nerves plays an important role in evoking protein secretion. Neuropeptide co-transmitters present in autonomic nerves also modulate secretion by salivary glands (Ekström 1999). Activation of beta adrenoceptors on salivary acinar cells increases intracellular cyclic adenosine monophosphate leading principally to stored protein secretion. Parasympathetic stimulation leading to muscarinic cholinergic receptor activation is linked to formation of inositol triphosphate and diacylglycerol and subsequent rises in intracellular calcium which open membrane ion channels, most notably apical chloride channels, leading to fluid secretion (Baum & Wellner 1999). Recent studies of salivary secretion have identified modulating roles for other signalling molecules such as cyclic guanosine monophosphate, cyclic adenosine diphosphate ribose and nitric oxide (Harmer *et al.* 2001). Similar mechanisms to those outlined above appear to operate in man.

Sjögren's syndrome (Vitali *et al.* 1993) and other inflammatory diseases affecting salivary glands such as parotitis (Ericson & Sjöback 1996) and HIV (Schiodt *et al.* 1992) have an associated salivary hypofunction, which although not life threatening can greatly decrease the patient's state of well-being. Within the affected salivary glands are large numbers of infiltrating inflammatory cells. The common assumption is that the salivary hypofunction is caused by destruction of parenchymal tissue. Certainly such destruction tends to occur progressively but there is evidence to suggest that there may be an inhibition of salivary secretion that precedes and then runs in parallel with tissue destruction (Humphreys-Beher & Peck 1999, Fox & Stern 2002). For example, in studies of cells isolated from labial salivary glands of subjects with Sjögren's syndrome agonist evoked ionic secretion (Dawson *et al.* 2001) and intracellular calcium signalling (Pedersen *et al.* 2000) were similar to cells from glands of healthy age-matched controls. However, it must be remembered that studies on minor salivary glands may not reflect the major glands which produce most saliva.

The rat submandibular gland has been used as a model to investigate the effects of obstructive diseases (Tamarin 1979, Martinez *et al.* 1982, Osailan *et al.* 2006), irradiation damage (Vissink *et al.* 1991), lipopolysaccharide (LPS)-induced endotoxaemia (Lomniczi *et al.* 2001) and intraductal adenovirus injection for gene therapy (Adesanya *et al.* 1996). Common to many of these models is an acute salivary hypofunction often developing within 24 h of the initial insult and resulting in salivary flows that can be just 20% of control values. It seems possible that inflammation causes salivary hypofunction. Following experimental ligation of the rat submandibular gland there is also an extensive glandular atrophy with infiltrates of neutrophils occurring in the early stage of obstruction (1–18 h) followed by a monocyte invasion, evident by 24 h (Tamarin 1979, Osailan *et al.* 2006). In the present study, we examined the secretory function of the rat submandibular gland following 24 h of ligation and explored possible mechanisms of salivary hypofunction.

## Materials and methods

### Ductal ligation and deligation

Twenty-three adult male Wistar rats (Harlan Labs, Loughborough, UK) weighing 275–325 g were placed under recovery anaesthesia (ketamine: 75 mg kg^−1^ and xylazine: 15 mg kg^−1^, i.p.) and a metal clip was placed on the submandibular duct through a small incision in the floor of the mouth. Following suturing animals were left to recover for 24 h before being placed under terminal anaesthesia (pentobarbitone: 48 mg kg^−1^ i.p., followed by chloralose: 80 mg kg^−1^, i.v.) for the procedures listed below. In three rats the parotid duct was ligated in the same way as for the submandibular duct. In a separate series of experiments, following 24 h of submandibular duct obstruction six rats were again placed under recovery anaesthesia to remove the metal clip obstructing the duct. Three days following the removal of the clip rats were placed under terminal anaesthesia to collect saliva as described below. In total 29 rats were used for the experiments described in this paper.

### Parasympathetic nerve stimulation

Under terminal anaesthesia three rats had the parasympathetic nerve supply to the submandibular gland electrically stimulated. The chorda lingual nerve was cut deeply and reflected onto the submandibular duct. Following cannulation of the duct with plastic tubing, posterior to the clip, both nerve and duct were placed in a bipolar electrode and stimulated at 2, 5 and 10 Hz (2 ms, 5 V) for 5 min each. Salivas were collected on ice into pre-weighed tubes to calculate salivary flow rates.

### Autonomimetic infusions

An intravenous cannula was inserted into femoral vein during induction of anaesthesia and this was connected to a calibrated syringe pump, as previously described (Proctor *et al.* 2003). Methacholine was continually infused at 12 *μ*g min^−1^ kg^−1^ body weight. Saliva samples were collected as described above from both left and right submandibular ducts simultaneously.

### Salivary analyses

Total salivary protein was measured by absorbance 215 nm assay, peroxidase was measured by fluorometric assay and salivary IgA by ELISA as described before (Proctor *et al.* 2003). Ions in diluted saliva (1 : 50 v/v deionized water) were measured using an ion selective electrodes (elit; Nico Ltd, Harrow, UK) calibrated against standard solutions. As the sodium electrode could be interfered by potassium ions, half of the potassium ion concentration was deducted from the sodium reading, as per manufacturer's instructions.

### *In vitro* cell analyses

Following ligation glands were removed from anaesthetized rats and digested by collagenase (5 mg/20 mL buffer) as previously described (Xu *et al.* 1996). Cells were stimulated either with methacholine (10^−5^m) or isoprenaline (10^−6^m) for 30 min under continuous oxygen at 37 °C as described previously (Carpenter *et al.* 2004). Cells were centrifuged, to separate the supernatant, and then homogenized in PBS containing a protease inhibitor cocktail (Calbiochem, Nottingham, UK). IgA and peroxidase were measured in cell supernatant and homogenate and expressed as percentage secretion [supernatant/(cell homogenate + supernatant) × 100].

Another aliquot of cells from the collagenase-digested glands were loaded with the calcium-sensitive dye Fluo 4 AM (4 *μ*m; Invitrogen, Paisley, UK) and stimulated with increasing doses of methacholine (10^−8^–10^−6^m), then ionomycin (13 *μ*m) and finally manganese chloride (2 mm) to calibrate levels of dye within each cell. Fluorescence levels were assessed in cells with an acinar morphology using a confocal microscope (TCS SP2, Leica, UK) – relative fluorescence levels are expressed relative to the maximum and minimum values obtained as (fluorescence − minimum)/(maximum signal − minimum).

### Histology

At the end of experiments submandibular glands were removed under anaesthesia, weighed and a section taken for fixation in formol/sucrose, the rest of the gland was frozen at −70 °C for further analyses. Fixed tissue was embedded in paraffin wax and 10 μm sections stained with haematoxylin and eosin for general morphology.

### Statistics

**Figure 2:**
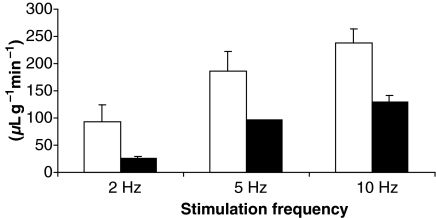
Parasympathetically stimulated rat submandibular flows from control (white) and 24-h ligated glands (black). The chorda lingual nerve supplying each gland was separately stimulated at 2, 5 and 10 Hz frequency for 5 min each. Mean flow rates (±SD) per gram gland wet weight for both glands were calculated using the unoperated gland weight as the ligated gland was oedematous (see Table 1), *n* = 3; *only at 10 Hz was a significant difference (*P* < 0.05) identified between control and ligated glands.

Results are expressed as mean ± SEM (except for Fig. 2 which is the mean ± SD). Multiple statistical comparisons were made using anova, whilst two-sample comparisons were made using Student's paired or unpaired *t* tests and *P* values <0.05 were considered significant.

**Table 1:** Submandibular gland weight changes for three types of experiments. Values are the mean (±SEM) values, *n* = 6 in all experiments. Controls were the contralateral unoperated submandibular gland to the 24-h ligated glands. Prior to stimulation salivary ducts were cannulated posterior to the ligature to avoid secretion against a blockage.

Experiment	24-h ligation without stimulation	Methacholine stimulation following 24-h ligation	Methacholine stimulation following 24-h ligation and 3 days of recovery
Control gland	0.22 ± 0.01	0.21 ± 0.01	0.25 ± 0.02
Ligated gland	0.26 ± 0.02	0.22 ± 0.01	0.18 ± 0.02
% Change	+19	+7	−29
*P* value	<0.01	<0.05	<0.01
Rat body weight (g)	292 ± 14	272 ± 8	307 ± 11

All animal procedures were conducted with approval of the local Animal ethics and welfare committee and under a home office project licence and all animals killed by an overdose of pentobarbitone.

## Results

### Gland weights and histology

Ligation of the rat submandibular duct induced a significant increase in gland weight but did not affect the contralateral unoperated gland (see Table 1). In the first group of rats, without methacholine stimulation, this increase was 19% greater than the control gland weight. In a second set of rats 24 h of ligation was followed by methacholine stimulation, in order to assess salivary function, in which the collecting cannulae were placed posterior to the ligation so that the gland was no longer obstructed. Following approximately 20 min of methacholine stimulation the previously ligated submandibular gland was just 7% larger than the control gland. There were no statistical differences in the weights of the control submandibular glands or total body weights between the first and second series of rats.

**Figure 1:**
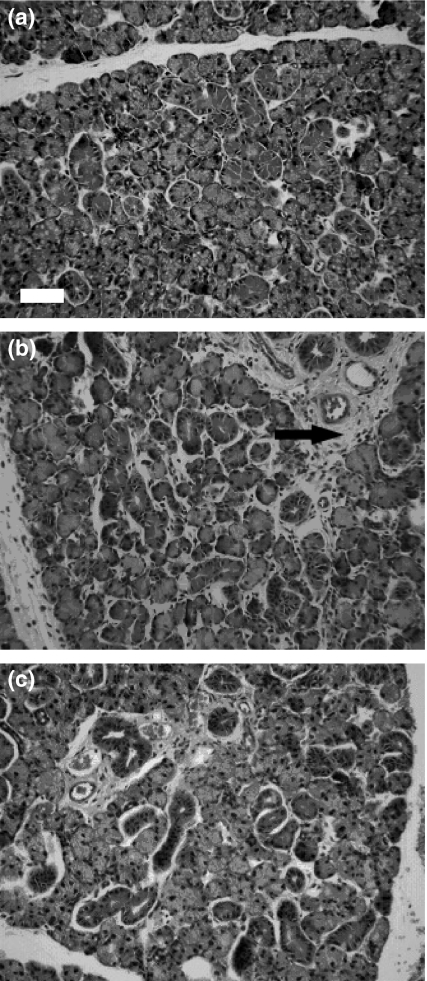
H & E staining of control unoperated rat submandibular gland (a), following 24-h ligation (b) and 3 days after removal of ligation (c). Ligated glands have large numbers of infiltrating inflammatory cells, readily apparent in the increased stroma and interstitial spaces (arrow) whereas control and recovered glands look normal with an absence of inflammatory cells. Scale bar = 30 μm.

The increased weight of the ligated gland was accompanied by changes in the histology as illustrated in Figure 1 by H&E staining of unstimulated control and ligated glands. Compared with the contralateral control submandibular gland the ligated gland appears less tightly packed and contains large numbers of infiltrating inflammatory cells. These cells are most prominent in the stroma of the gland although they are also present within the interstitial space between acini and ductal units. The infiltrating cells appear to be mostly neutrophils and macrophages (based on cell size and shape) although no detailed analysis was attempted. In addition, blood vessels and salivary striated duct lumena appeared dilated in the ligated gland compared with the control gland.

### Salivary secretion in vivo

At all frequencies of parasympathetic nerve stimulation salivary flows from the ligated side were reduced by at least 50% compared with the control and at 10 Hz this difference was statistically significant (Fig. 2). Flow rates for both the control and the ligated gland were calculated per gram tissue weight using the wet weight of the control gland as the ligated gland was oedematous. Whole-body methacholine stimulation was subsequently used in order to bypass any effects of ligation on the nerve–epithelial cell junction. Methacholine stimulation also revealed salivary hypofunction in the 24 h ligated salivary gland (Table 2). A methacholine dose 12 μg kg^−1^ body weight min^−1^, producing control gland flows equivalent to 2–5 Hz parasympathetic stimulation, demonstrated even greater salivary hypofunction – flows on the ligated side were just 20% of the control gland. Similar results were also obtained following ductal ligation of the parotid gland (123 ± 5 vs. 44 ± 15 μL g^−1^ min^−1^, control vs. ligated, mean ± SD, *n* = 3). Analysis of the methacholine-induced submandibular salivas revealed that in addition to reduced saliva production there was also a similarly reduced output (concentration × flow rate) of total protein and IgA, although the reduced IgA output was not as great as for total protein it was still significantly reduced (Table 2). The concentrations of ions in the same samples of saliva also indicated reduced ion reabsorption as their concentrations were greater in samples from the ligated gland.

**Table 2:** Methacholine (12 μg kg^−1^ min^−1^) stimulated salivary flow rates and components collected simultaneously from the control and the contralateral 24-h ligated gland, *n* = 6. Mean ± SEM flow rates for both glands are calculated using the control gland weight.

	Flow rate (μL g^−1^ min^−1^)	Total protein output (mg g^−1^ min^−1^)	IgA output (μg g^−1^ min^−1^)	Chloride ions (mmol L^−1^)	Sodium ions (mmol L^−1^)
Control	157 ± 19	1.20 ± 0.18	0.93 ± 0.23	9.43 ± 1.00	23.9 ± 5.76
Ligated	32 ± 7	0.18 ± 0.02	0.26 ± 0.05	46.89 ± 11.72	54.8 ± 6.29
*P* value	<0.001	<0.001	<0.05	<0.05	<0.05

### Salivary protein secretion in vitro

**Figure 3:**
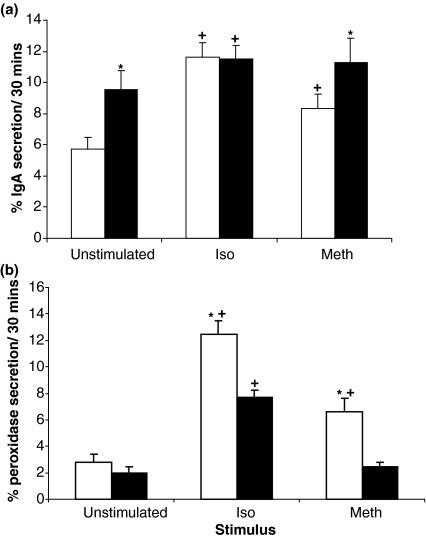
Percentage IgA (a) and peroxidase (b) secretion from submandibular cell preparations of the unoperated control (white columns) and the contralateral gland that had previously been ligated for 24 h (black columns). The results are expressed as the mean ± SEM (*n* = 19 from four separate ligation experiments for IgA and *n* = 10 from two experiments for peroxidase) secreted as a percentage of the total present in cells and medium; *P < 0.05 compared with cells of the contralateral gland for the same treatment, +*P* < 0.05 compared with the unstimulated cells from the same gland.

Cells from the unoperated submandibular gland secreted significantly more IgA and peroxidase (Fig. 3) with isoprenaline (10^−6^m) or methacholine (10^−5^m) than in the absence of the autonomimetics. However, cells from the previously ligated gland produced mixed results. Isoprenaline still caused a significant increased secretion of IgA and peroxidase compared with the unstimulated cells from the ligated gland although the protein secretion response to methacholine was no longer significant.

### Cellular calcium signalling

**Figure 4:**
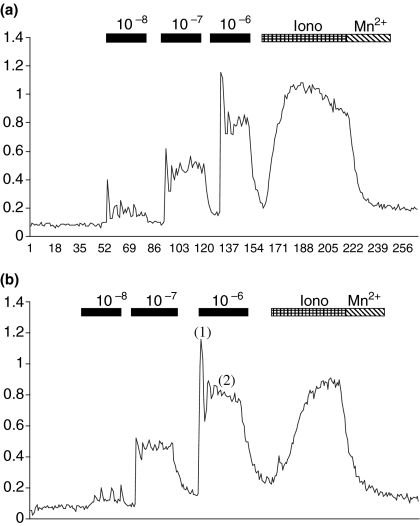
Relative fluorescence against time (s). Cells from control (a) and 24-h ligated glands (b) were stimulated with increasing doses of methacholine (10^−8^–10^−6^m). Cells were then incubated with ionomycin (iono) and then manganese chloride (Mn^2+^) to elicit the maximum and minimum fluorescence signal. During stimulation by methacholine a biphasic response was seen comprising a peak (1) and plateau (2) phase.

Despite altered protein secretion in response to the cholinergic agonist methacholine in acinar cells from the ligated gland intracellular calcium concentrations in response to methacholine were similar for cells from the control and previously ligated glands (Table 3). Figure 4 shows typical traces for one acinar cell from the unoperated gland and previously ligated submandibular gland. A typical biphasic trace was observed for cells from both glands. Cells from the ligated gland tended to have higher levels of calcium (Table 3), this was only significantly different during 10^−8^m methacholine stimulation.

**Table 3:** Intracellular calcium levels (as relative fluorescence) in cells from control and ligated glands examined *in vitro*. Cells were loaded with the dye Fluo 4-AM and then monitored by confocal microscope for intensity changes following incubation with different concentrations of methacholine. At the end of experiments cells were incubated with ionomycin to induce the maximum fluorescence. Thus relative fluorescence was determined as (fluorescence − minimum)/(maximum signal − minimum). The results are the mean ± SEM of five different ligation experiments with two preparations of each gland containing 5 to 10 cells and were compared by non-paired *t*-tests.

		10^−8^m		10^−7^m		10^−6^m	
				
	Baseline	Peak	Plateau	Peak	Plateau	Peak	Plateau
Control	0.09 ± 0.01	0.47 ± 0.04	0.29 ± 0.02	0.73 ± 0.04	0.47 ± 0.03	0.90 ± 0.03	0.62 ± 0.02
Number	58	44		58		58	
Ligated	0.11 ± 0.01	0.57 ± 0.04	0.38 ± 0.03	0.78 ± 0.04	0.54 ± 0.03	0.97 ± 0.02	0.64 ± 0.02
Number	59	37		59		59	
*P* value	n.s.	n.s.	*P* < 0.05	n.s.	n.s.	n.s.	n.s.

### Recovery from ductal ligation

**Figure 5:**
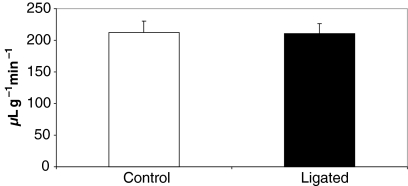
Methacholine-induced salivary flow rates from glands 3 days after the removal of a 24-h ligation experiment (black column) compared with the contralateral control gland (white column). Although the recovered gland was 30% smaller (see Table 1) the secretory output per gram gland wet weight was the same. The flow rates were not statistically different from the control gland flow rates shown in Table 2.

Three days following removal of the ductal ligature methacholine-stimulated salivary flows from the previously ligated glands were improved compared with 24 h ligated glands but was still less than the contralateral side when expressed as total flow per gland. The previously ligated glands were now approximately 30% lighter than the unoperated control gland (Table 1). When expressed per gram of gland weight the flows were the same (Fig. 5). The histology of these glands indicated a reduced inflammatory cell infiltrate and a normal morphology of the parenchyma (see Fig. 1c).

## Discussion

The 24-h time point for ductal ligation was chosen as a previous study (Martinez *et al.* 1982) had shown this time point to be the earliest at which salivary hypofunction develops. Unlike the Martinez study (Martinez *et al.* 1982) we used an intra-oral ligation technique that avoids any damage to the chorda-lingual nerve supplying parasympathetic impulses into the salivary gland. By avoiding any possible damage of the chorda-lingual the complications of a denervation-induced atrophy are avoided (Harrison *et al.* 2001). In addition, the 24-h time point precedes any obvious acinar protein degranulation that is often regarded as the starting point of atrophy (Ohlin & Perec 1967, Scott *et al.* 1999, Harrison *et al.* 2000, 2001, Osailan *et al.* 2006). The ligated glands were oedematous which was reduced following methacholine stimulation suggesting it was partially caused by the build up of saliva within the gland in addition to inflammation-related oedema. The effect of the methacholine stimulation also coincided with the removal of the blockage (to collect saliva) which may have prevented more saliva being pushed back into the gland.

Acinar cell fluid secretion is caused by the secretion of chloride which is later reabsorbed by ductal cells (in particular striated ducts) further down the secretory tree. Ion reabsorption is generally regarded as occurring at one rate so that the slower the flow of saliva the greater the reabsorption of ions. Despite a much lower salivary flow rate salivas from the ligated gland had greater concentrations of sodium and chloride, in agreement with Martinez *et al.* (1982). Thus ductal function was also inhibited following 24-h ligation. The reasons or even possible mechanisms for this are unclear at present and require further investigation.

Removing the ligated and unoperated submandibular glands from rats to form enzymatically dispersed cellular units enabled a more detailed analysis of acinar function. Protein secretion and changes in intracellular calcium reflect the two main signalling cascades in salivary acinar cells (Huang *et al.* 2001, Melvin *et al.* 2005). For protein secretion peroxidase was used as a marker for acinar storage granule secretion (Proctor *et al.* 2000) whilst IgA, though not stored, is transported through acinar and ductal cells by the polymeric immunoglobulin receptor and its rate upregulated by autonomic stimulation (Carpenter *et al.* 1998). Cells were incubated with the autonomimetics isoprenaline, for a β-adrenergic stimulus, and methacholine, as a cholinergic stimulus. Although β-adrenergic stimuli is the main stimulus for protein secretion *in vivo* (Proctor *et al.* 2003) and *in vitro* (Baum & Wellner 1999) cholinergic stimuli can cause some protein secretion (Scott *et al.* 1999). Isoprenaline caused a significant secretion of IgA and peroxidase by cells from unoperated control glands, as shown previously (Baum & Kuyatt 1981, Anderson 1986, Carpenter *et al.* 2004). Cells from ligated glands still secreted significantly more IgA and peroxidase above unstimulated cells although the increased peroxidase secretion was less than by cells from the unoperated gland. The increased unstimulated IgA secretion by cells from ligated glands compared with unoperated control glands presumably reflects increased pIgR translocation to the apical membrane although the reasons for this are unclear. The methacholine-evoked secretion (which better relates to the *in vivo* secretion results) of peroxidase and IgA secretion was affected – levels were no longer different to secretion by unstimulated cells. The difference between IgA and peroxidase secretion from the same cells may well relate to the different storage and transport mechanisms of these proteins, for a review see Proctor (1999). The loss of methacholine-induced protein secretion from cells *in vitro* is in contrast to the changes in intracellular calcium in acinar cells caused by methacholine stimulation. Cells from control and ligated glands both produced a typical biphasic response – the first peak relating to calcium release from internal stores, the plateau reflecting influx of calcium from outside the cell (Gallacher & Smith 1999). All cells analysed responded to the two higher doses of methacholine but ∼15% fewer cells from ligated glands responded to the lowest dose of methacholine compared with cells from the control gland. This may reflect a reduction in muscarinic receptors noted in a previous study of cells from 24-h ligated rat submandibular glands (Martinez *et al.* 1982). The results from these *in vitro* studies suggest that salivary acinar cells are not severely inhibited, as they appear to be *in vivo*. In the process of collagenase digestion salivary cells, which are larger than inflammatory cells, may become separated from inflammatory cells as they were washed and centrifuged several times. Thus we speculate that the influence of the inflammatory cells on salivary cells may have been reduced in these *in vitro* cell preparations. However, a caveat with all *in vitro* studies, it cannot be excluded that further disruptions may occur downstream from the intracellular calcium signalling events to cause the altered protein and fluid secretion seen *in vivo*.

Although salivary secretion from the ligated/deligated glands was reduced compared with the unoperated side upon *in vivo* methacholine stimulation, when salivary flows were expressed per gram of secretory tissue the rates of saliva secretion had recovered to the same level as the control. Thus the mechanism of acinar salivary hypofunction is reversible and the majority of loss of secretory function following 24 h of ligation was not due to cell death caused by either apoptosis or necrosis as both are irreversible. Interestingly rates of apoptosis in our study, as assessed by TUNEL and the expression of M30, were similar in 24-h ligated glands to unoperated glands and in agreement with a previous study (Takahashi *et al.* 2000) which indicated significantly increased TUNEL rates only occurred after at least 2 days of ligation. Thus it is unclear what has caused the 30% loss of gland weight and this will require further investigation. In other models exhibiting salivary hypofunction, such as LPS, adenovirus instillation and irradiation authors also suggest that cellular destruction appears insufficient to cause the large initial decreases in salivary flow (Vissink *et al.* 1991, Adesanya *et al.* 1996, Scott *et al.* 1999, Lomniczi *et al.* 2001).

Bradykinins are the product of kallikreins acting on kininogens and can exert a direct effect on salivary secretion (Vo *et al.* 1983). Tissue kallikreins are stored in large amounts in the granular tubules and are predominantly secreted into saliva. To determine a possible role of tissue kallikreins in salivary hypofunction, acute blockage of the parotid gland was performed. As found with the submandibular gland, a 24-h blockage of the parotid gland caused a 70% decrease in stimulated salivary flow. As the parotid gland contains relatively little kallikrein it appears unlikely that stored submandibular kallikreins have a large role in hypofunction, although further experiments are required to eliminate (plasma-derived) bradykinins.

By examining salivary function following 24 h of ductal ligation we have a useful model of inflammation and salivary hypofunction. Unlike the LPS and adenovirus models there are no infection-related mechanisms of secretory inhibition and by examining salivary function before 2 days of ductal ligation (using the intra-oral ligation technique) we have avoided any obvious atrophy, although this cannot be completely eliminated. The use of whole-body methacholine stimulation has bypassed any effect of oedema on the nerve–salivary cell neuro-effector junction. In contrast to the extreme salivary hypofunction demonstrated *in vivo* cells from the ligated glands have a normal biphasic intracellular calcium response to a cholinergic agonists – the main stimulus for fluid secretion and still secreted a significant amount of protein in response to β-adrenoceptor stimulation – the main stimulus for protein secretion. Alterations in methacholine-evoked protein secretion suggest some changes have occurred in the acinar cells and this will be further investigated. It seems logical to assume that the cause of salivary hypofunction relates to the inflammatory infiltrate as both develop with time. Our next goal is to eliminate the inflammation during ligation to see if salivary hypofunction still develops.

## Conflicts of interest

The authors have no conflicts of interest.
